# Zn-Containing Membranes for Guided Bone Regeneration in Dentistry

**DOI:** 10.3390/polym13111797

**Published:** 2021-05-29

**Authors:** Manuel Toledano, Marta Vallecillo-Rivas, María T. Osorio, Esther Muñoz-Soto, Manuel Toledano-Osorio, Cristina Vallecillo, Raquel Toledano, Christopher D. Lynch, María-Angeles Serrera-Figallo, Raquel Osorio

**Affiliations:** 1 Colegio Máximo de Cartuja s/n, Faculty of Dentistry, University of Granada, 18071 Granada, Spain; toledano@ugr.es (M.T.); mvallecillo@correo.ugr.es (M.V.-R.); emsoto@ugr.es (E.M.-S.); mtoledano@correo.ugr.es (M.T.-O.); cvallecillorivas@hotmail.com (C.V.); rosorio@ugr.es (R.O.); 2 Independent Research Scholar, Av. Fuerzas Armadas n1, 18014 Granada, Spain; mtoleosorio@gmail.com (M.T.O.); rtoleosorio@gmail.com (R.T.); 3 Restorative Dentistry, University Dental School & Hospital, University College Cork, Wilton, T12 E8YV Cork, Ireland; 4 Oral Surgery Section, Faculty of Dentistry, University of Sevilla, Avicena s/n, 41009 Sevilla, Spain; maserrera@us.es

**Keywords:** zinc, membranes, bioactivity, guided bone regeneration, mechanical, microscopy, antibacterial, cytotoxicity

## Abstract

Barrier membranes are employed in guided bone regeneration (GBR) to facilitate bone in-growth. A bioactive and biomimetic Zn-doped membrane with the ability to participate in bone healing and regeneration is necessary. The aim of the present study is to state the effect of doping the membranes for GBR with zinc compounds in the improvement of bone regeneration. A literature search was conducted using electronic databases, such as PubMed, MEDLINE, DIMDI, Embase, Scopus and Web of Science. A narrative exploratory review was undertaken, focusing on the antibacterial effects, physicochemical and biological properties of Zn-loaded membranes. Bioactivity, bone formation and cytotoxicity were analyzed. Microstructure and mechanical properties of these membranes were also determined. Zn-doped membranes have inhibited in vivo and in vitro bacterial colonization. Zn-alloy and Zn-doped membranes attained good biocompatibility and were found to be non-toxic to cells. The Zn-doped matrices showed feasible mechanical properties, such as flexibility, strength, complex modulus and tan delta. Zn incorporation in polymeric membranes provided the highest regenerative efficiency for bone healing in experimental animals, potentiating osteogenesis, angiogenesis, biological activity and a balanced remodeling. Zn-loaded membranes doped with SiO_2_ nanoparticles have performed as bioactive modulators provoking an M2 macrophage increase and are a potential biomaterial for promoting bone repair. Zn-doped membranes have promoted pro-healing phenotypes.

## 1. Introduction

**Figure 1:**
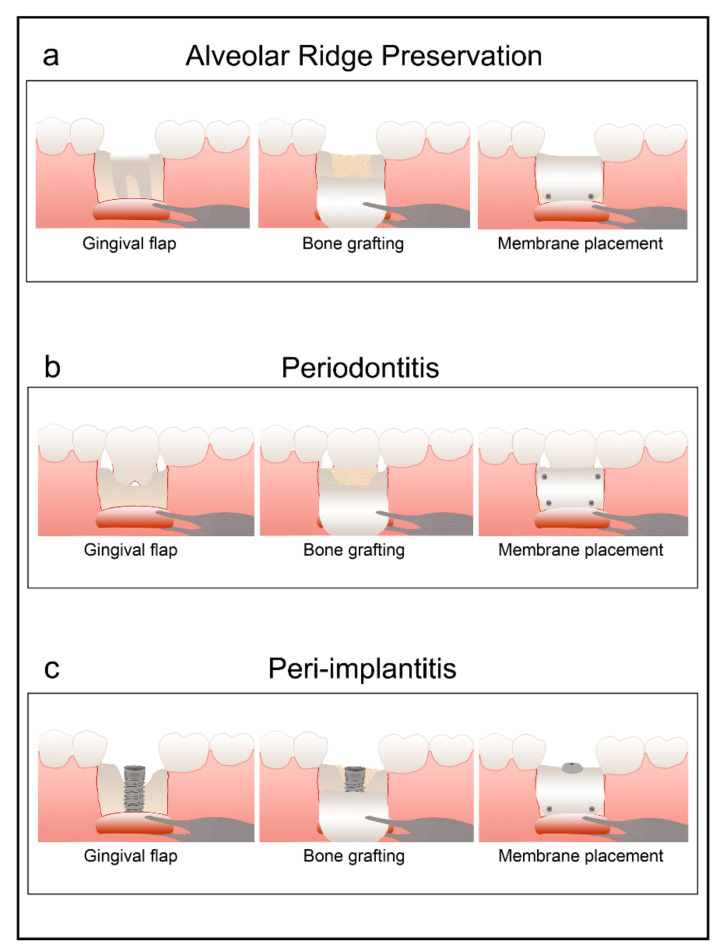
Illustration representing the surgical protocol for alveolar ridge preservation with membranes after tooth extraction (**a**). Use of membrane in regenerative procedures for periodontal defects (**b**). Membranes for peri-implantitis regeneration (**c**). In all cases, membranes are stabilized with mini-screws and pins to the surrounding bone.

The development of oral implantology has generated an increasing interest in procedures aimed toward maintaining and preserving both bone and soft tissue after tooth extraction. Followed by tooth loss, a process of bone remodeling occurs causing the reduction of socket dimensions [1]. This damage is more pronounced and close to 50% during the first three months [2]. To reduce the impact of this remodeling during alveolar healing, various techniques and materials have been studied to increase localized bone volume, making these regenerative procedures a main part of temporary implant therapy [3,4] (Figure 1a). Periodontitis is a bacterial-mediated inflammatory process that affects both the gingiva and anchoring tissues of the tooth; if not treated, it may progressively destroy periodontal tissues, eventually resulting in tooth loss [4].

Bone tissue engineering [5] has focused on the development of materials with the purpose of supporting bone formation, reducing the impact of bone remodeling and stimulating bone regeneration [6]. To achieve these goals, several biomaterials have been employed, including autologous bone, bone substitutes (allografts, xenografts, and alloplasts), blood [7] and active barrier membranes [8]. In this line, it has been recently shown from a clinical standpoint that quercetin-zinc complex incorporated into polycaprolactone/gelatin nanofiber can promote bone regeneration [9]. Anorganic bovine-derived hydroxyapatite/cell-binding peptide grafts have been proposed to treat intrabony defects in conventional periodontal surgery [10]. In order to provide bone cells within the secluded space for bone regeneration, a barrier membrane is placed to protect the clot within the socket and to isolate it from adjacent connective tissue [6,11]. This procedure is known as Guided Bone Regeneration (GBR). GBR can be applied in the treatment of both periodontitis (Figure 1b) and peri-implantitis (Figure 1c). Nevertheless, with this regenerative technique, long-term maintenance of enough space to provide sufficient bone is under question [12,13].

Available GBR membranes can be classified according to their resorbability characteristics. Collagen membranes are the most representative, characterized by their high biocompatibility and biodegradability [14]. However, they lack sufficient mechanical strength to remain stable over time and spatially protect the defect area [15]. When using non-resorbable membranes, the newly formed bone might have a protection from physiological stresses, which is required for remodeling and maturation. Non-resorbable membranes include expanded, high-density, titanium-reinforced polytetrafluoroethylene (PTFE), and titanium mesh [12]. PTFE is a synthetic polymer consider the gold standard against resorbable membranes. One drawback in the use of this type of membrane is the necessity for its removal with a second-stage surgical procedure [12]. Poly(ε-caprolactone) pellets and gelatin type B from bovine skin were used to construct an electrospun membrane with improved hydrophilicity and better cell-membrane response [4]. Polymethylmethacrylates (PMMA) are non-resorbable polymers that improve the mechanical performance of hydrophilic structures [16,17,18]. These biocompatible and biostable polymers exhibit weak interactivity with tissue cells and favor a biological response based on the formation of fibrotic tissue [19,20]. The bioactivity of these materials is mainly related to the absorption capacity of calcium, phosphate and bone-related proteins [20]. The failure or success of the implanted device is determined by a bioprocess recovery, highly influenced by adsorption of proteins [21], as they perform like bioactive molecules [22]. Proteins are constructed by amino acids functional groups as carboxyl (COOH) and amino (NH_2_). It has been demonstrated that cell differentiation and adhesion, and protein adsorption [23] may be altered by the modification of materials surfaces with those groups of amino acids [24]. A new proposal for composite membranes results from mixing (MMA)_1_-co-(HEMA)_1_ and (MA)_3_-co-(HEA)_2_ by electrospun [20]. These membranes perform as scaffolds that attempt to mimic the extracellular matrix (ECM) in both shape and size [20]. One of the main advantages of these types of membranes is the possibility to get their functionalization. This feature poses a pivotal breakthrough in current tissue engineering. Adding some extra components allows the modification of the physical and biological properties to improve the interaction with the surrounding tissue [20,25]. 

Loading membranes with bioactive compounds can accelerate the bone formation process [26]. Recent studies have proposed to include some metallic materials, such as Fe- and Mg-based alloys, and more recently Zn-alloys [14] to biodegradable matrices. Zn is an essential mineral that is vital for routine skeletal growth [9]. Zn can be found in the human body, being present in all tissues and more than 200 types of enzymes [14]. Zn participates in numerous physiological and metabolic processes, playing a fundamental role in the immune and nervous systems [27]. Electrospun polymer-based membranes loaded with ZnO have shown enhanced cell proliferation/wound healing [4]. Incorporation of Zn into a membrane for GBR favors the proliferation of osteoblasts [25], triggers bone neoformation and inhibits bacterial biofilm formation [28,29,30]. In addition, animal studies using Zn-doped nanostructured membranes show faster bone healing and regeneration [9,30].

The present manuscript is a narrative review where previously published scientific evidence is summarized and synthesized. Nevertheless, this review does not follow rules about the search for evidence, and decisions about the importance of included manuscripts were not systematically performed. Systematic reviews, due to their methodology design, provides stronger evidence than narrative reviews, but it is difficult to perform a systematic review when the literature available and knowledge on the issue in question is scarce. In contrast to systematic reviews, narrative reviews can address one or more questions, and the selection criteria for inclusion of the articles may not be specified explicitly. Subjectivity in study selection is the main weakness ascribed to narrative reviews that potentially leads to biases. Certainly, the quality of a narrative review may be improved by borrowing from the systematic review methodologies that are aimed at reducing bias in the selection of articles for review and employing an effective bibliographic research strategy [31]. The aim of the present study was to state and assess the potential effectiveness of doping the membranes for GBR with zinc compounds in order to improve bone remineralization and regeneration.

## 2. Materials and Methods

### 2.1. Question Addressed by This Review 

Which benefits can be attributed to Zn-doped membranes in GBR? 

### 2.2. Literature Search 

An exploratory narrative review was conducted. Electronic databases, such as PubMed, Scopus, Web of Science, MEDLINE, DIMDI and Embase, were used to undertake the literature search. Additionally, after conducting a hand-searching of the literature, the references of similar and related studies were also included. Bone formation (MeSH Terms), bone regeneration (MeSH Terms), osteogenesis (MeSH Terms), “Zinc”, “Zn”, “Zinc-doped”, “membrane”, “membranes”, “matrix”, “scaffolds”, “guide bone regeneration”, “GBR”, “alveolar ridge preservation” and “periodontal regeneration” were the main search terms. There was no time limit established and English-written articles were only selected. The included articles in the present narrative review were those in which the main objective was to study Zn-containing membranes and establish their benefits if applied in oral surgery and periodontology. After deep reading of the included articles, performance of membranes containing Zn was evaluated by means of bioactivity and remineralization assessment, antibacterial effects and physicochemical properties of the membranes, microstructure, mechanical properties and so cells cytotoxicity and proliferation onto membranes. 

### 2.3. Eligibility: Inclusion and Exclusion Criteria for Studies

Inclusion of an article was based on the following inclusion criteria: Studies assessing the effectiveness of Zn-containing membranes by comparing changes in bioactivity, degree of remineralization and regeneration, antibacterial potential, physicochemical-mechanical properties, microstructure, cytotoxicity and cells proliferation. Then, the present pre-selected studies were evaluated according to the following exclusion criteria:

1. Insufficient information on type or zinc content.
2. Duplicate studies, commentaries and letters to the editor.

### 2.4. Preparation of Zn-Doped Membranes for Guided Bone Regeneration

#### 2.4.1. Zn Phosphate Mineralized Collagen Membrane

Polyglycolic acid/trimethylene carbonate (PGA/TMC) copolymer membrane. Resolut Adapt LT (W.L. Gore & Associates, Inc., Flagstaff, AZ, USA), and bovine-derived collagen membrane, BioMend Extend (Zimmer Dental, Carlsbad, CA, USA) [32], were cut into 1 cm^2^ sections and mineralized. Two methods: (i) the precipitation method, consisting in incubating each section in 20 mL zinc phosphate solution (potassium phosphate and zinc acetate) for 3 and 7 days at 37 °C; and (ii) the microwave method developed by LeGeros et al., [32,33], immersing each section in 20 mL of zinc phosphate solution and placing it in the microwave oven, activated on medium power until the solution evaporates completely. The membranes were then rinsed with double distilled water and allowed to air dry.

#### 2.4.2. Composite Membrane of Zn Bioactive Glass and Polylactic Acid 

Bioactive Glass and poly-L-D-L-lactic acid (PLDLA) were used. To prepare zinc-containing bioactive glass (ZnBG) powders, Oh et al., [34] set precursors in appropriate concentrations (70SiO_2_–25CaO–5ZnO) that were dissolved in ethanol–water solvent which contained HCl, and stirred for 6 h. After 24 h aging at 40 °C, the solution was dried for several days at 70 °C. The gelled product was heat-treated to 650 °C, held there for 3 h, and then cooled to room temperature. To prepare ZnBG powders, the resulting powders of heat-treatment were pounded and sieved down to 45 µm, dissolved in tetrahydrofuran (THF) and mixed with PLDLA at 4% (*w*/*v*) for elaboration of composite membranes with polylactic acid (PLA). The glass/PLDLA composition was fixed at ~3/7 by weight to maintain an important mechanical flexibility and a great amount of glass. Finally, to form a membrane of ~150 mm thickness, the solution was agitated for 24 h and put into a teflon mold to evaporate the solvent. After incubation of the membranes in a simulated body fluid, the surface morphology and the crystalline phase were evaluated with field emission scanning electron microscopy (FESEM) and X-ray diffraction (XRD), respectively. The mechanical properties were measured by applying a tensile load at a speed of 1mm/min using an Instron 3344. 

#### 2.4.3. Composite Membrane of Poly(ε-caprolactone), Gelatine Type B from Bovine Skin and ZnO Nanoparticles 

Poly(ε-caprolactone) pellets and gelatine type B from bovine skin and ZnO nanoparticles were used to construct an electrospun membrane. The polymers were dissolved in hexafluoruro-2-propanol to produce a 10 wt% solution (100 mg mL^−1^) stirred overnight and added at different concentrations relative to the total polymer weight. The obtained materials were collected and dried under vacuum for at least 48 h to complete removal of solvents [4]. The membranes were observed by FESEM, energy dispersive X-ray spectroscopy (EDX), transmission electron microscopy (TEM) and Fourier transform infrared spectroscopy (FTIR). Contact angle, mechanical properties, antimicrobial activity and cytotoxicity tests were also performed. A mixture containing CaO: CaHPO4: ZnO = 0.7: 2: 0.3 (molar ratio) was used by Chou et al., 2014 [26] to produce the zinc hydroxyapatite (Zn-HAp) powder samples, which were ground in an agate centrifugal ball mill. Gelatine blocks were completely dissolved in 60 °C water for 3 h at a 5% (wt) concentration, cooled to 40 °C in ice, mixed with Zn-HAp powders at 70 mg/mL concentration and then were 12 h air-dried to produce the membranes. Before being freeze-dried for 12 h, membranes were allowed to swell for 1 h in 4 °C distilled water. Finally, the membranes were cross-linked by heat at 150 °C for 5 h. Powder XRD analysis was used to evaluate Zn-HAp powders. The thickness and the morphological features of the surface were observed under SEM. Two derivatives of Zn(II) quercitin [(Zn(quercitin) H_2_O_2_) (Zn + Q), and Zn(quercitin) (phenanthroline) (Zn + Q(PHt)] were synthesized and characterized using UV-Visible spectrophotometer and Fourier Transform-IR spectroscopy. The UV-Visible absorption and IR spectra prove the B-ring chelation of the flavonoid quercetin to Zn(II) rather C-ring chelation. At the molecular level, Runx2, mRNA and protein, ALP and type 1 collagen mRNAs, and osteoblast-specific microRNA, pre-mir-15b were examined using real-time RT-PCR and Western blot assay [9]. 

#### 2.4.4. Polymeric Membranes with Zinc Ions Complexation 

**Figure 2:**
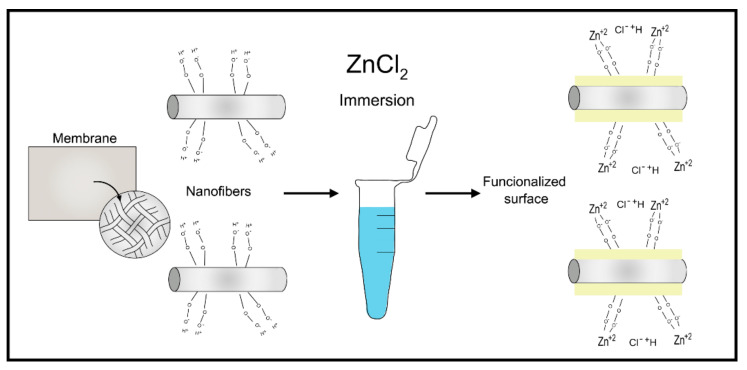
The carboxyl-terminal on polymeric Zinc-doped membranes surfaces, after their immersion on ZnCl_2_.

**Figure 3:**
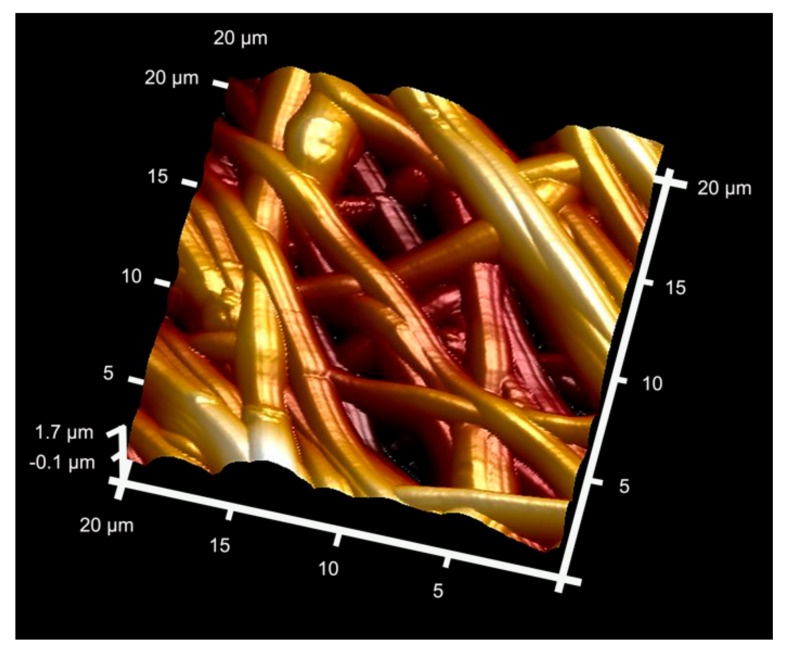
AFM image of the surface’s Zn-matrix. Overlapped and randomly distributed nanofibers may be observed. Spotty nanodeposits, at high magnification, are distributed onto the Zn-fiber surfaces.

The membranes were prepared with a commercial polymeric mixture (PolymBlend^®^) [18]. PolymBlend^®^ is made of a blend of two copolymers with high molecular weight: (i) [(MA)_3_-co-(HEA)_2_] (average Mw 2000 kDa, PDI < 1.5) and (ii) [(MMA)_1_-co-(HEMA)_1_] (average Mw 200 kDa, PDI < 2.5). A non-woven nanofiber material with adequate physicochemical and mechanical properties was produced by electrospinning. Before electrospinning, purification of cured copolymers (three dissolutions in acetone and precipitation of monomer in water) was used to discard unreacted monomers. After the creation of the nano-structured membranes (NMs), the nanofiber membrane was heat-treated and immersed it in hot water (60 °C) for 4 h. Protein adsorption was determined for zinc-doped NMs-NH_2_ (aminated) and NMs-COOH (carboxylated) membranes. Then, the membranes were loaded, by chemical functionalization, with zinc (Zn-NM) (40 ppm, for 3 d, pH 6.5) and incubated for 3 days at room temperature [28]. The attained ion complexation values were 3 μg Zn/mg of membrane. To reach equilibrium of adsorption of metal ions, they were shaken in different solutions of ZnCl_2_ (composed of zinc 40 ppm and pH 6.5) (Figure 2) and then, suspensions were centrifuged for 60 min (two cycles, 12.000 rpm). After centrifugation, the matrices were separated from the supernatant [29]. For the surface preactivation, a sodium carbonate buffer solution (333 mM and pH 12.5) was used to react with Zn-matrix and OH-matrix surfaces for 2 h and then gently washed with water. Therefore, carboxyl groups were arranged on their surfaces due to the partial hydrolysis of ester bonds [17]. A new polymer blend was recently synthesized starting from (MA)_3_-co-(HEA)_2_/(MMA)_1_-co-(HEMA)_1_ but further comprises 5% (wt) of SiO_2_ nanoparticles (SiO_2_-NPs), suspending them in the electrospinning solution (Figure 3). In the membrane, SiO_2_-NPs were homogenously dispersed and also trapped in the whole fiber volume forming a solid solution (composite) [6]. To functionalize the Zn-loaded membranes, the ability of carboxyl groups to complex divalent cations was used. Two types of membranes were manufactured: (1) SiO_2_-NP doped membrane (HOOC-Si-Membrane) and (2) SiO_2_-NP doped membrane functionalized with Zn (Zn-HOOC-Si-Membrane).

#### 2.4.5. Membranes Composed by Zn-Li-Mg, Zn-Li and Zn-Li-Ag Alloys 

Zhang et al., 2019 [14] prepared pure Zn, Li, Mg and Ag (>99.9%) membrane. To formulate Zn-0.8%Li-0.2%Mg (wt), Zn-0.8%Li (wt), and Zn-0.8%Li-0.2%Ag (wt) alloys. Ingot dimensions (Ø60 mm × 200 mm) were achieved through melting samples in graphite crucibles at 580 °C in a resistance furnace under an Ar atmosphere and then cast into a 550 °C cylindrical steel mold. Inductively coupled plasma atomic emission spectrometry (ICP-AES) was used to analyze alloy composition. To homogenize the structure, it was annealed at 400 °C for 24 h and cooled in water. Finally, sheets with a thickness of 0.1 mm were obtained by placing in a box furnace at 250 °C and then hot rolling them with intermediate annealing. 

## 3. Results

### 3.1. Antibacterial Effects of Zn-Loaded Membranes 

The minimum inhibitory concentration differed in all concentrations tested for function of the bacteria species [4]. All the membranes containing distinct concentrations of ZnO showed antibacterial activity against the studied bacteria, with relevant inhibition zones ranging from 6 to 15 mm in diameter, being the caprolactone 30% the highest effective.

To determine the antibacterial effect, the mean bacterial colony forming between 4 and 48 h for zinc-loaded membranes was evaluated and then compared with those non-mineralized by immersing each membrane in an *Actinobacillus actinomycetemcomitans* culture. Comparing the mean colony forming unit value for mineralized collagen membranes between 4 and 24 h showed more than a 3-fold drop at 24 h mean in antibacterial effect. The difference in the overall mean colony forming unit value between mineralized collagen membranes and non-mineralized collagen membranes was statistically significant [32]. The antibacterial effects of non-mineralized and Zn phosphate mineralized membranes were also evaluated at different incubation times, and decreased at 48 h. Among the membranes tested, mineralized collagen membranes achieved better antibacterial effects than non-mineralized and Zn phosphate mineralized membranes. The non-mineralized membranes attained significantly higher counts of bacterial colonization compared to Zn phosphate mineralized membranes. 

Periodontal biofilms were studied by Bueno et al., 2020 [28] after 12 h, 48 h and 72 h by scanning electron microscopy (SEM). After 12, 24, 48 and 72 h, DNA of growth biofilms was isolated and then quantified using PCR for the six bacterial species used. Each DNA sample was analyzed in duplicate and the correlation between quantification cycle (Cq) values and CFU mL-1 were generated through a software. As shown by SEM, NMs doped with zinc resulted in structured biofilms from 12–72 h. Quantitative evolution of the bacterial load revealed a lower total number of bacterial on NMs doped with Zn (Zn-NMs) compared to control biofilms up to 48 h. At 72 h, this effect was lost, and a significantly higher bacterial count was found on Zn-NMs. 

### 3.2. Physicochemical and Biological-Related Properties of Zn-Loaded Membranes

Due to the physiological conditions, the release of zinc ions from the membranes was studied by the authors in an acetate base (pH 4.5) and a phosphate base solution. Crystallographically, by powders XRD analysis, the peak patterns verified signature peaks associated with amorphous HAp. Mass spectroscopy showed Zn concentration to be approximately 5%. Morphologically, crater-like structures characterized the surface of membranes. The thickness of the membranes resulted to be roughly 100 µm [26]. The release pattern of Zn showed an initial burst release in the first 12 h before reaching an equilibrium concentration after 40 h; the levels remained constant until the 72-h mark. The acidic nature of the solution degraded some parts of the membranes [26]. In Toledano et al., 2020 [20], both NMs-NH_2_ and NMs-COOH membranes attained similar fibronectin (Fn) and plasma proteins (PP) adsorption. NMs-COOH membranes adsorbed significantly more albumin than NMs-NH_2_. NMs-NH_2_ membranes adsorbed significantly more fibrinogen (Fg) than NMs-COOH.

### 3.3. The Effect of Zn-Loaded Membranes on Bioactivity and Bone Formation

The precipitation of calcium phosphate on the PLDLA-ZnBG started at day 3 and was pronounced at day 7, covering the surface almost completely. The crystalline phase developed was characteristic of apatite [34].

**Figure 4:**
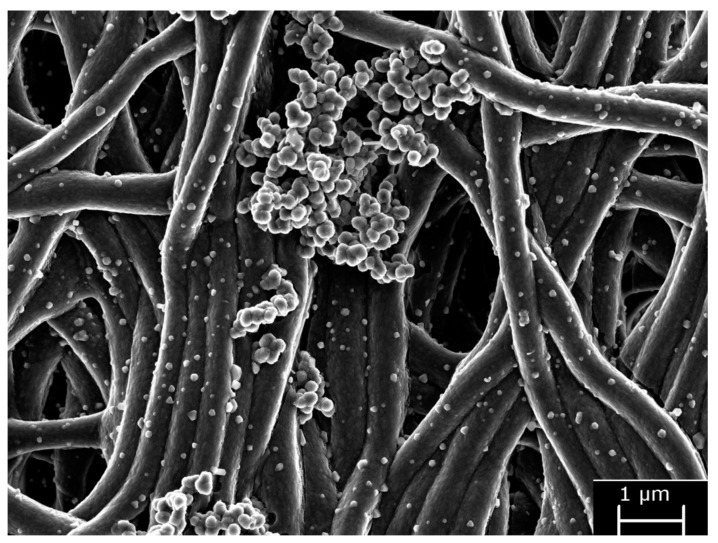
FESEM micrographs of a Zn-doped membrane after immersion in SBFS. Nanofiber diameter is about 500 nm, and fibers lost the smooth appearance of their surface. Nanodeposits of mineral (100 nm) are randomly distributed onto the nanofiber surfaces. Numerous agglomerations of other spherical nanocrystals (bigger than 200 nm) are identified onto the Zn-matrix surface.

In vitro tests of bioactivity showed mineral nanodeposits of 100 nm placed on the surface of the fibers in a random way [18]. Calcium and phosphorus were found on the surface of the nanofibers that composed the membrane, in the EDX spectrum. Numerous agglomerations of more than 200 nm of spherical ZN nanocrystals were detected (Figure 4). Furthermore, calcium, phosphorus and zinc appeared after the elemental analysis of crystal agglomerations. 

In vivo test used thirty, non-adult, Wistar rats due to their adequate size for evaluating membranes and response to implanted biomaterials. These 15-week-old animals were randomly divided into experimental groups. Every 2 weeks until 6 weeks, new bone formations were evaluated with a software based on the CT scans (40 µm/pixel voxel size) [26]. Zn-doped membranes achieved statistically more new bone formation in comparison to the unloaded groups. At 6 weeks, the bone recovery at the defect site from Zn-doped membranes was 75% approximately. At 6 weeks, the newly generated bone in the Zn-doped group reached a maturation level identical to that of the surrounding host bone. At both Toledano et al., 2019 and 2020 [6,30] microcomputed tomography (micro-CT) was used to analyze rabbit skulls. The average bone density (HU) was assessed by a PMOD software, and Bone*J* (Image*J* plugin) was employed to evaluate bone architecture. Six white, New Zealand-breed rabbits composed the sample with the same characteristics regarding age, 6 months, and weight, 3.5–4 kg. Von Kossa morphometric study of the regenerated bone defects was carried out to visualize the mineralized bone, obtaining information about osteoid surface (OS), bone surface (BS), percentage of osteoid surface (OS/TS) and bone perimeter (BPm), bone thickness (BTh), and percentage of bone area (BS/total surface (TS)). To observe the deposition of calcein into the newly deposited bone matrix, fluorescence images were obtained. Osteoblasts, osteocytes, and blood vessels were evaluated at toluidine blue (TB) images. Osteoclasts, macrophages (M1 and M2 and ratio M1/M2), were also assessed. 

**Figure 5:**
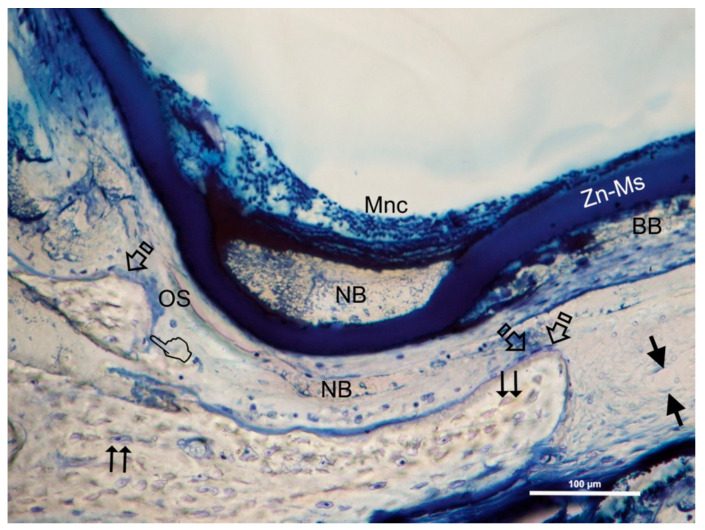
Bone histology image obtained by dye with toluidine blue after using Zn-NMs, in experimental animals after their healing time. Few isles of newly formed trabecular bone (NB) were observed at both side of Zn-NMs. Single arrows point the presence of osteoblasts; double arrows mean osteocytes and pointers indicate osteoclast. Face arrows mean blood vessels. Bony bridging (BB), mononuclear cells (Mnc) and osteoid (Os) may be observed.

**Figure 6:**
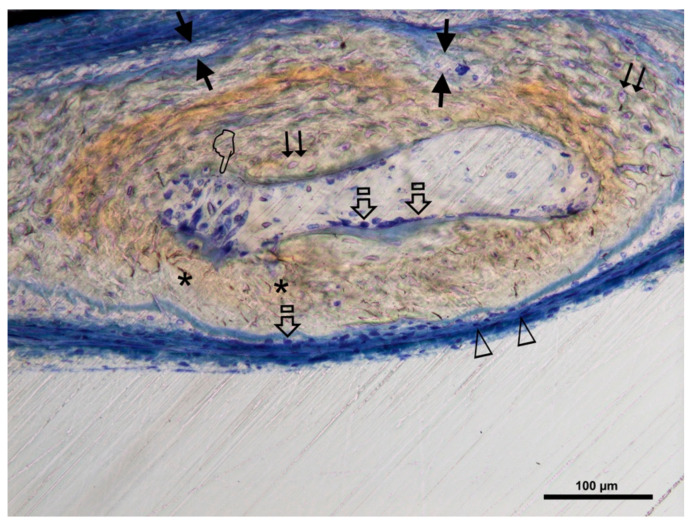
Bone histology image obtained after using Zn-NMs dye with toluidine blue in experimental animals to visualize mineral bone after their healing time. Single arrows point the presence of osteoblast with typical cuboid shape. Double arrows indicate osteocytes, pointers mean osteoclast and faced arrows point blood vessels. Asterisk are located close by canaliculi. Arrow heads signal the presence of osteoid.

**Figure 7:**
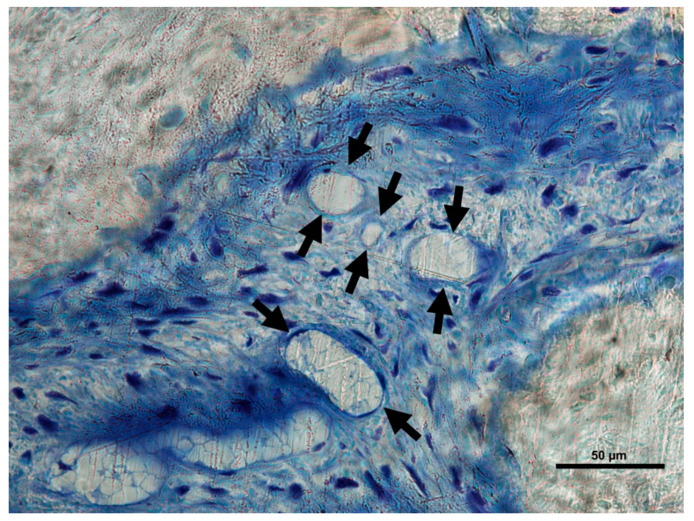
Bone histology image obtained in samples treated with Zn-loaded membranes by coloration with toluidine blue in experimental animals. At high magnification, vessels are clearly visualized (faced arrows) promoting and maintaining bone maturation.

**Figure 8:**
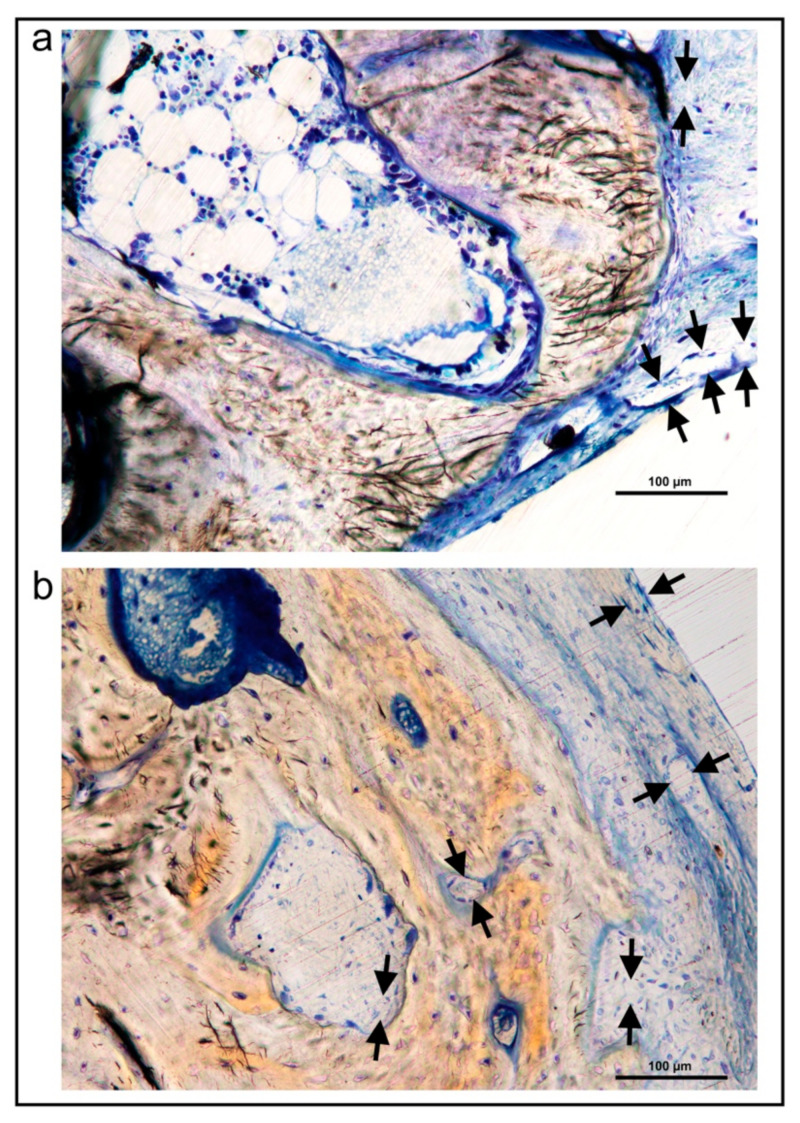
Bone histology images obtained by dye with toluidine blue after using Zn-loaded membranes in experimental animals. Blood vessels (faced arrows) that connect bone marrow directly with the blood supply can be easily observed (**a**,**b**).

The Crop analysis performed in Toledano et al., 2019 [30] showed that compared to the control, Zn-NMs produced a higher BS. Zn-NMs presented a greater number of branches and junctions, trabecular bone, when the skeleton analysis was performed. When observing the center of the defect (culture 150) with micro-CT, Zn-NMs showed higher characteristics of Euler and bone spatial connectivity compared with control. The analysis skeleton through micro-CT permitted to observe higher nodes, branch points and longer branches, increased new bone formation (BS/TS ratio), greater BTh and higher OS than the control group. Interstitial connective tissue was visible in samples treated with Zn-NMs. TB staining showed reactionary bone formed with lacunae bridging of osteocytes on the Zn-NMs. Observing Zn-NMs, new bone formation could be seen both below and outside the membrane, while on both sides of it few isles of new bone were observed. These isles presented, from the limits of the defect, a stockade of osteoblasts and osteoid (Figure 5 and Figure 6). No signs of inflammation infiltrate could be observed. While the osteocytes count was similar between the different groups, osteoblasts were found in greater numbers in Zn-NMs (Figure 5 and Figure 6). These osteoblasts appeared immediately on contact with the membrane in some fields. Many large vessels could be detected in samples treated with Zn-NMs (Figure 7 and Figure 8). 

The chick embryo chorioallantoic membrane (CAM) assay demonstrated that the angiogenic parameters were increased by the (Zn + Q(PHt)) complex. Meanwhile, the (Zn + Q(PHt)) complex showed significant activity and thereby this complex was further examined for the bone tissue activity by incorporating the complex into a nanofiber through electrospinning method [9].

### 3.4. Analysis of the Microstructure through Microscopic Characterization 

Microstructure was examined by SEM and TEM, whose specimens thinned 80 µm; the constituent phases were identified by XRD [14]. Homogeneous (from 56 to 1184 nm) and heterogeneous (from 93 to 2223 nm) diameter distribution was observed in caprolactone and caprolactone-gelatin-based membrane, respectively [4]. The NPs showed, basically, a rod-like profile. The fibers showed some swollen areas and distinct areas embedded with ZnO NPs. All membranes attained C-H bond of saturated carbons and the ester-carbonyl group (-CO stretching).

Surface nanoroughness (SRa) was examined, in Osorio et al., 2017 [18], Osorio et al., 2020 [29] and Toledano-Osorio et al., 2021 [25], using atomic force microscopy (AFM). A specific software (Nanoscope) was employed to measure fiber to fiber distance and fiber diameters with Image*J*. Attached to an EDX, FESEM was used to perform an additional surface characterization to analyze membranes and look for phosphate and calcium deposits. XRD was used to detect crystal formation and its main components. Mean and standard deviation of nanofiber sizes ranged from 302.40 (SD 19.13) to 312.72 (SD 28.66) nm from AFM observations [18]. The fiber size was approximately 300 nm in diameter, through FESEM analysis (Figure 4). Microfibers had a size ranging from 2.05 (SD 0.30) to 1.97 (SD 0.18) nm. Fiber to fiber distances were in a wide range, between 110 and 11.5 μm. Zn-loaded tissues showed a SRa of 108.40 (SD 16.17) nm. Zn-Li and Zn-Li-Ag alloys were composed of a Zn matrix with a secondary LiZn_4_ phase, whereas the Zn-Li-Mg alloy consisted mainly of a Mg_2_Zn_11_ phase, in the proposal of Zhang et al., 2019 [14]. Ag was completely dissolved in the Zn matrix. Microstructure of alloys containing Zn showed a fibrous structure along with the rolling direction, where the grains and the secondary phase were crushed, lengthened into a fibroid, and distributed along the rolling direction. In the Zn-Li-Mg alloy, an intermediate phase (Mg_2_Zn_11_) with an elliptical shape uniformly appeared in the Zn matrix. The secondary phase was confirmed to be Mg_2_Zn_11_ and was not observed for the Zn-Li-Ag alloy. Zn-Li showed fine grains and small subgrains, implying that recrystallization occurred during the rolling process. A large number of spherical particles were homogeneously distributed inside the grain, attributed to the secondary LiZn_4_ phase. The Zn-Li alloy had precipitation size of approximately 10–30 nm, with a high density. 

### 3.5. In Vitro Mechanical Behavior of Zn-Doped Membranes 

PLDLA-ZnBG membranes showed similar tensile strength that the undoped membranes and so high degree of elongation and flexibility [34]. 

**Figure 9:**
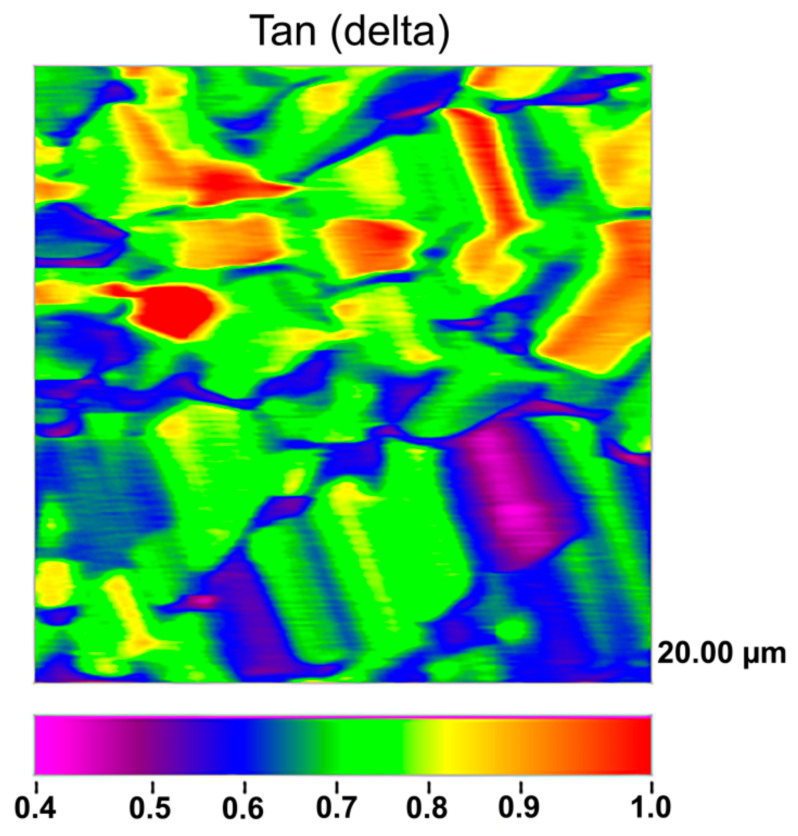
Nano-DMA analysis, on scanning mode, of the surface’s Zn-matrix. The property map corresponds to the tan delta values.

Under a hydrated condition of the specimens, Hysitron TI Premier nanoindenter equipped with a nano-DMA software (DMA-III) was employed to conduct nanomechanical property mappings, in Osorio et al., [18,29]. Loss modulus (E), complex modulus (E*), tan delta (δ), and storage modulus (E’) (GPa) were calculated. Zn-matrices achieved a complex modulus of 17.40 (5.36) GPa, a loss modulus of 6.60 (1.44) GPa, a storage modulus of 13.29 (5.25) GPa and a tan delta δ of 0.65 (0.15) in Osorio et al., 2017 [18] (Figure 9). The Zn-Li alloy showed a high yield strength (183.5 ± 5.3 MPa), ultimate tensile strength (238.1 ± 4.7 MPa) and elongation (75 ± 6%) [14].

### 3.6. Cells Cytotoxicity and Proliferation

To assess the in vitro cellular behavior, rat bone marrow mesenchymal stem cells (rBMSCs) were used in Oh et al., [34]. Cell viability and morphology were determined using an ELISA reader and confocal laser scanning microscopy, respectively. Cells were viable, with high total protein content, for PLDLA-ZnBG membranes, showing well-developed cytoskeletal filaments in active stretching [34]. There was a significant alkaline phosphatase rise and activity with increasing culturing time, after the presence of Zn [14]. Zn content also promoted the growth of osteocalcin. The mineralization quantified demonstrated a significantly higher Ca level on the PLDLA-ZnBG membranes. Zn-Li-Ag alloy was innocuous and satisfactory for bone tissue engineering in the study attained by Zhang et al., 2019 [14].

Münchow et al., (2015) [4] reported cytocompatibility in all membranes. Nevertheless, the presence of a higher concentration of NPs led to a brief toxic effect on the cells, being the caprolactone-gelatine presentation the material having the highest cytotoxic potential.

Primary cultures of human oral mucosa fibroblasts [18] were obtained to determine cell viability by three different techniques: (1) cell death, quantifying DNA liberated to the culture media; (2) the lactate dehydrogenase (LDH) assay, detecting the amount of LDH which is released by cells with damaged membrane; and (3) fluorescence-based method to evaluate cell membrane integrity and cytoplasmatic esterase function, quantifying the number of green (live) and red (death) cells (LIVE/DEAD). Liberated DNA to the culture media was found to be very low in Osorio et al., [18]. Human fibroblasts viability stated above 80% in zinc-loaded groups. All the experimental groups attained high levels of cell viability. Zn-loaded membranes produced a negligible degree of cytotoxicity, and a dose-dependent effect was not noticed after applying any test. Osorio et al., 2020 [29] used Alamar-Blue^TM^ assay to determine cell proliferation. FESEM was utilized to study cell morphology.

## 4. Discussion

There are multiple factors to be studied on the present membranes. Multi-parametric characterization comprises the study of surface characteristics such as porosity, pore size and roughness, mechanical properties of the surface, in vitro static bioactivity and biological characterization by cell culture methods are required [11,25,35].

### 4.1. Antibacterial Effects of Zn-Loaded Membranes 

The antibacterial effect of zinc phosphate mineralized collagen and zinc phosphate mineralized copolymer membranes has been demonstrated [32]. This antibacterial effect on mineralized membranes of zinc phosphate, collagen or copolymer, which results from the inhibition of bacterial colonization, favors the regeneration and healing of both soft and hard tissues [36]. Increased concentration of ZnO did not inhibit zones against *Porphyromonas gingivalis*, but against Fusobacterium nucleatum that antibacterial activity was greater at 10,000 µg/mL [4]. NPs can produce reactive oxygen species which may penetrate the cell wall and affect bacterial integrity. The higher the amount of NPs incorporated, the greater the availability of active compound to diffuse into the agar and inhibit bacterial growth. Even more, gelatine can interact with the cell membrane promoting its destruction/death. Polymeric NMs loaded with Zn were able to alter the kinetics and development of in vitro biofilms [28]. However, this antibacterial effect decreased when the biofilms reached the stationary phase (72 h). The loss of antimicrobial effect is explained by the full coverage of the NMs surface by non-vital bacterial cells [28]. In addition, it has been previously claimed the importance of modifying the membrane’s structure by adding metal components as zinc to achieve antibacterial effect [25].

### 4.2. Physicochemical and Biological-Related Properties of Zn-Loaded Membranes

For the development of a biodegradable Zn-HAp membrane, zinc hydroxyapatite powder was incorporated into gelatine solutions [26]. The membrane production process was relatively simple, and the result was a biomaterial with a rough surface, which would encourage cell activity. This Zn-HAp membrane has shown the potential to be used in GBR, allowing the release of ions (zinc, phosphate and calcium) on the defect, stimulating bone regeneration in rat calvaria. Some of the demonstrated characteristics of the membrane are cellular adaptation, immobility, differentiation, and the permeation of vital nutrients and growth factors for GBR [26,37,38,39]. It has been speculated that when osteoclasts adhere to the rough surface of the membrane and were liberated into the localized area, zinc ions, along with calcium and phosphate, would be released. Hence, they would depress osteoclast bone resorption by inducing osteoclast apoptosis, promoting osteoblast differentiation and proliferation [26]. It was shown that bone formation was faster compared to samples testing collagen membrane or in specimens without membrane. In the Zn-NMs group there was a recovery and regeneration of the defect from the second week. This process continued throughout the study period, demonstrating a more complete and unified remodeling mechanism [26]. The staining analysis of hematoxylin and eosin did not reveal any signs of fibroblast infiltration. The newly deposited bone on the membrane showed well-distributed bone formation with similar maturation and thickness patterns than the original bone. This process of bone creation occurred before the Zn-NMs reabsorption. Osteogenesis happens along the surface of Zn-NMs probably due to the higher concentration of Ca ions compared to the collagen membrane.

At polymeric carboxylated membranes immersed in Kokubo solution, calcium phosphate deposits were created. However, a lack of bioactivity was stated for the aminated membranes, due to calcium ions in SBF which are apt to be trapped by the double scissors of COO-function. Then, the still bounded calcium ions attracted negatively charged phosphate ions from the solution [40] to form a columnar Ca framework in calcium phosphate deposits. Furthermore, it has been reported that HAp formation was successfully achieved with weaker acidic -PO_4_H_2_ and -COOH, but not with -OH and -CH-CH_2_ [41]. Notably, the -COOH end group appeared to provide the optimal surface for nucleation and growth of biomimetic HAp [20].

### 4.3. The Effect of Zn-Loaded Membranes on Bioactivity and Bone Formation

The use of zinc in bone tissue engineering has become increasingly important, as more and more studies have shown the influence of Zn-HAp on the osteoblast proliferation and bone regeneration. It has been demonstrated that if osteoblasts are exposed to Zn ions, the production of transforming growth factor-beta (TGF-β) is favored and increases the production of osteoprotegerin (OPG) [26]. OPG is responsible for inhibiting osteoblast differentiation and function by acting as a decoy for osteoclast receptor activators of nuclear factor kappa-β ligand (RANKL). Chou et al., [26] after evaluating in an animal model, a membrane functionalized with zinc, confirmed the barrier properties and the response of the host tissue to the use of Zn-NMs. Histological evaluation of the samples showed good cell separation and new bone formation. The bone formation around Zn-NMs was well distributed throughout the membrane and defect with a maturation and thickness similar to the original bone. Zn-NMs achieved osteogenesis thanks to the presence of calcium ions in higher concentration on its surface. These properties confirm the intrinsic and important role of zinc in bone tissue formation demonstrating the relationship that does exist between Zn, bone remodeling and regeneration processes. Then, the influence of Zn-NMs can be potentially advantageous for clinical applications [26]. 

The in vitro bioactivity of a material is defined as the ability to create bony bonds with the host bone. It can be predicted from the formation of calcium phosphate (Ca/P) deposits on the surface after simulated body fluid solution (SBFS) immersion [42,43]. Ca/P deposition can be explained as a surface phenomenon [44]. Osorio et al., 2017 [18] demonstrated that after immersion in SBFS, zinc is found in the hard tissues favoring the precipitation of Ca/P deposits and the generation of HAp nanocrystals. Zinc deposits on tissues helped for phosphate group binding. These phosphate groups have poorly coordinated oxygens, resulting in reactive surfaces that will attract calcium ions from SBFS [45]. The biomimetic Ca/P deposition, inspired by the natural biomineralization process, is considered a coating method even capable of generating periodontal regeneration. The presence of HAp determines bone formation and osteoconduction since this molecule favors the formation of bone apatite-like materials, being able to promote cells recruitment [46]. Osteoblasts, cementoblasts, periodontal ligament and pulp cells stimulated with extracellular Ca^2+^ and PO_4_^2−^ increased bone morphogenetic protein-2 (BMP-2) mRNA expression [8,47,48]. Increases in Ca^2+^ concentration is related to higher levels of the fibroblast growth factor-2 (FGF-2) gene and protein expression, in the periodontal ligament [49]. FGF-2 is responsible for neoformation, has a mitogenic effect on cementoblasts and cells of the periodontal ligament, and improves the regeneration of periodontal tissue. It also accompanied the formation of new bone and cementum with functionally oriented periodontal ligament fibers [49]. The main advantage of the chemical formulation of the presented matrices is that they possess a high calcium-binding affinity, which is essential for cell differentiation and bone regeneration. Moreover, direct covalent binding and immobilization of a high load of almost any type of biomolecule (enzyme, growth factors, antibody, antigen, antibiotic…) at their surfaces have been encountered [18].

**Figure 10:**
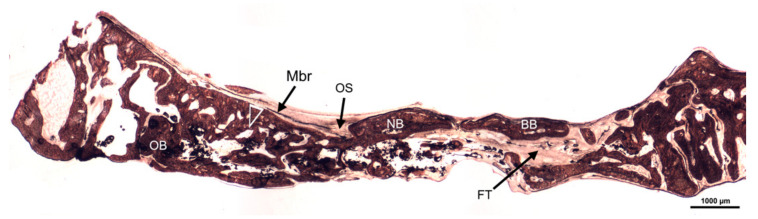
Bone histomorphometry obtained by dye with Von Kossa silver nitrate stain to visualize formed mineralized bone in experimental animals at six weeks of follow-up, around Zn-loaded membranes. Trabecular bone formations were formed along the margin of calvarial defect and within the defect (arrow head). Bony bridging (BB), Fibrous tissue (FT), Membrane (Mbr), Osteoid (OS), Old bone (OB).

**Figure 11:**
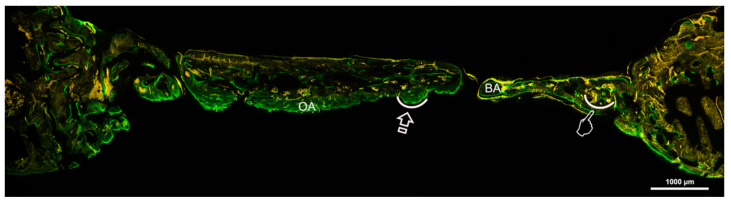
Bone histology obtained by fluorescence with calcein at the region of interest to see mineralized bone, at six weeks of healing time after using Zn-loaded membranes. Pointers indicate bone perimeter and arrows signal osteoid perimeter. Bone area (BA), Osteoid area (OA).

Precipitation of calcium and phosphate on the matrix surfaces was observed in zinc-loaded specimens [18]. The increase in structural indexes, through tomographic analysis that was observed after Zn-NMs application [30] resulted in the replacement of older, overly mature bone with younger and more resilient bone [50]. Osteoid or bone matrix that will be, but not yet, mineralized [51] showed higher surface than in the control group when Zn-NMs were used, a sign of young new bone formation [52]. The pattern of the tissue appeared composed by Zn-NMs in close contact with the newly formed bone and with osteoid. The significant increase of mineralized bone matrix excluding osteoid [51] (Figure 10) was related to an increase in osteoblasts prompted by all membranes in comparison with the control group, especially when Zn-NMs were used. Newly formed bone, associated with multiple interconnected ossified trabeculae, was observed directly in contact with the Zn-NMs surfaces in the regions that showed successful bone conduction [53]. The newly formed bone was continuous in some exploration sections, forming bony bridges from the margin of the defect and without invasion of soft tissue (Figure 5 and Figure 10). The changing osteonal morphology is probably related to the maturation and maintenance of the bone vasculature, which is a clear sign of remodeling, based on both nutrient supply and cell recruitment [54] (Figure 8 and Figure 9). Osteogenesis is always preceded by angiogenesis [55]. Therefore, it is logical to think that the incorporation of Zn provides a greater regenerative efficiency during bone healing (Figure 6 and Figure 11). Not only the osteogenesis was determined, but also the improved biological activity according to the number of osteoblasts when Zn-NMs were used. 

The zinc ion is considered a promising osteoimmunomodulator acting on macrophage polarization and conditioning the differentiation of osteogenic cells [56]. Bone formation is a sign that the membranes are capable of causing osteoblast growth and differentiation to fill, completely or partially, the intracortical pores with nucleating clusters. These mineral deposits will subsequently fuse, resulting in amorphous Ca/P and finally apatite crystals [57], to reactivate the cells of the bone lining into bone-forming osteoblasts. The connectivity of the pores described in these membranes [18] could influence the possibility that a greater number of osteoblasts can penetrate the porous structure [58]. Zn has been shown to protect both collagen from degradation [16] and seed crystallite-sparse collagen from remineralization. Zn also influences the signaling pathway and stimulates the metabolic effect of hard tissue mineralization [59]. Zn-NMs act as bioactive modulators of the signals communicated to the underlying defect. These results provide evidence that Zn-NMs may be useful for tissue engineering but should be viewed with caution until corroborated in clinical studies.

### 4.4. Analysis of the Microstructure through Microscopic Characterization

The membrane of ZnBG-PLDLA was shown to be flexible when handled, suggesting the possible applicability for a dental biomaterial within a periodontal pocket [34]. The increase viscosity of the gelatine-based solutions plus caprolactone may lead to the occurrence of thicker fibers compared to those gelatine-free [4]. Addition of higher amount of ZnO NPs increases the electrical conductivity of the polymer, reducing the self-repulsion tension and the elongation forces, hence reducing fibers width. Nanometric porosity ranging from 50 to 500 nm selectively enhances protein adsorption (including fibronectin and vitronectin), contributing to cell attachment [18,60]. Cells growing on membranes containing pores between 5 and 8 μm showed increased osteogenic differentiation [61]. These pore sizes are frequently encountered in the presented tissues, being approximately 30% of pores on each tissue. Dynamic recrystallization occurred during the rolling processing in the study attained by Zhang et al., 2019 [14]. The average grain size of the Zn-Li-Ag alloy (2.3 µm) was smaller than that of the Zn-Li alloy (4.1 µm), indicating that adding an Ag alloying element refined the grains of the Zn-Li alloy. The high density of fine dispersive precipitates could impede the dislocation movements, leading to a strength increase for alloy. The precipitates also showed a regular interface [14]. 

### 4.5. In Vitro Mechanical Behavior of Zn-Doped Membranes 

ZnBG-PLDLA membranes retained sufficient level of mechanical strength and flexibility for space maintenance and handling [34]. They also inducted a Ca/P mineral phase on the surface within a relatively short period. Blending gelatine in junction with caprolactone increases the crystallinity and mechanical strength of fibers [4]. Crosslinking reactions contributed to improved mechanical properties and degradation resistance. The ZnO incorporation revealed a decrease of both the tensile strength and the Young’s modulus. Storage moduli of Zn-matrices are within the range of the nanoindentation moduli of calcified trabecular bone, which are about 15 GPa [18,62]. These values highly differ from the storage modulus calculated for highly cross-linked collagen scaffolds, which is around 1 GPa [63]. The importance of these results is supported by a recent finding stating that substrate stiffness can modify cell behavior and cells may probe and respond to mechanics in fibrillar matrices [64]. They presented the highest ability to store potential energy which is released after deformation. Dissipation of energy within the structures is of prime importance in dynamic systems [65,66], such as the oral function, where oral structures require damping to absorb shock waves and alleviate stresses. Hence, improving damping characteristics becomes imperative for enhancing their robustness and force resistance. During contact, low-modulus materials lead to stress concentration resulting in transfer of energy (without dissipation) from the tissues to the adjacent structures. Zn-matrices attained adequate tan *δ* values (0.6), being more favorable to cell spreading as recently published by Baker et al., 2015 [64]. In Zhang et al., 2019 [14], the Zn-Li alloy showed a ductile fracture surface with dimples and a few shear lips, indicating that the plasticity of the Zn-Li alloy could be theoretically improved. The Zn-Li (Mg, Ag) alloys prepared exhibited high tensile properties (especially plasticity), superior to those of reported Zn-based alloys such Zn-Mg or Zn-Ca alloys. The excellent mechanical properties could be ascribed to the precipitation strengthening, the grain refinement and to the dynamic recrystallization [14].

### 4.6. Cells Cytotoxicity and Proliferation 

All membranes, in Münchow et al., (2015) [4], presented cell viability higher than 50% and was optimal even when ZnO content up to 15%, thus confirming the biocompatible nature of the ZnO-incorporated membranes. Nevertheless, incorporation of a higher amount of NPs provoked a slight toxic effect on the cells. At low concentration (<5 wt.%), ZnO was not harmful to cells. When mesenchymal stem cells were cultured on the ZnBG-PLDLA membranes, the cells were shown to adhere favorably and continued to actively proliferate with time, having adequate growth kinetics. The osteoblastic development was satisfactory, enhancing the mineralization process of the ECM of cells developed on the substrate [34]. Matrices of Zn-loaded membranes were found to be non-toxic to fibroblast in all the assays performed by Osorio et al., 2017 [18], where Zn-loaded scaffolds presented a very low cytotoxic effect. The morphology of L929 cells after 5 days of incubation in different extract mediums with concentrations of 10, 50, and 100% showed that L929 cells were healthy and exhibited a flattened spindle shape, similar to that of negative control [14]. Osorio et al., 2017 [18] stated that the inclusion of calcium and zinc ions on the membranes’ surface are not cytotoxic, as it was highlighted above. However, metals accumulation in the body may also be considered. It has been reported for the different studied GBR Zn-containing membranes that the biological significance of a metal concentration depends on the specific tissue in which the metal is deposited, and toxicity cannot be predicted from total metal burden in the organism [67]. Recent studies have shown that zinc exhibits better in vivo degradation behavior without leaving voluminous corrosion products that are hard to be eliminated by human body [68]. According to ISO 10993-5: 1999, the cytotoxicity of these extracts of Zn-Li-Ag alloy was of Grade 0–1. In other words, the Zn-Li-Ag alloys are innocuous and satisfactory for bone tissue engineering [14]. It can be concluded that the incorporation of metallic nanoparticles to the biomaterial did not exert cytotoxicity and avoided adverse effects on the organisms. Quercetin-zinc complex (Zn + Q(PHt)) incorporated into polycaprolactone/gelatin nanofiber acted as a pharmacological agent for treating bone associated defects and guided bone regeneration [9]. Histology studies revealed that the (PCL/gelatin/Zn + Q(PHt)) showed in-vivo biocompatibility [9].

The Zn-containing membranes have not shown zones of inhibition against certain bacteria (*Porphyromonas gingivalis*) [4]. The antibacterial activity of ZnO-loaded membranes was found to be dose-dependent (≥5 wt.%) [69]. Increased concentration of ZnO could reduce bacterial load and counts of the tested bacterial species but when the biofilms reached 72 h phase the antimicrobial effect of the Zn-NM decrease. It has been explained that the full coverage of the NMs surface by non-vital bacterial cells may have helped for this decrease of antimicrobial effect [28]. The incorporation of high ZnO NPs content might decrease the fiber thickness [4]. This slimming process also might hinder the cell colonization process and angiogenesis for a proper guided bone regeneration [6]. ZnO nanoparticles possess high surface energy, so they tend to agglomerate when mixed in higher concentrations, forming clusters along the fiber and, consequently, causing a significant morphological change. These characteristics may also negatively influence cell proliferation, cell colonization process and angiogenesis for a proper guided bone regeneration. However, the combination with gelatin allows the incorporation of a greater amount of ZnO without damaging changes in morphology and microstructure [4]. On the other hand, simple Zn-Li alloys create thicker grain size precipitates within the sheets formulated by Zhang et al., (2019) [14]. Nevertheless, they proposed to include an Ag alloying group aimed to refine the grains of the Zn-Li alloy [14]. Besides, the plasticity of the Zn-Li alloy has been demonstrated to be scarce and, at least in theory, should be improved by trying to incorporate other Zn-based alloys [14]. 

ZnO nanoparticles are not soluble in water. At higher ZnO contents (e.g., 30 wt.%) the surface energy of the membrane could be reduced, thus resulting in lower wettability [4]. Optimal wettability is important at GBR, since the membranes are placed in direct contact with fluids present in the surgical site [70]. It has been also pointed out that the ZnO inclusion may show a decrease of the tensile strength and the Young’s modulus, but the manufacturers offset this drawback by incorporating crosslinking reactions [4]. ZnO NPs inclusion on the same polymers increased membranes’ cell cytotoxicity in a dose-dependent manner obtaining a reduction in cell viability up to 50%; therefore, ZnO concentration should be maintained under certain limitation [4].

In order to improve and expand the clinical applications of functionalized nanostructured membranes, other substances and molecules may be used for loading. The possibility that the matrices presented could be chemically functionalized and loaded with almost any type of biomolecule (enzyme, growth factors, antibody, antigen, antibiotic...) on their surfaces, presents a wide field of study [18,25]. However, it is important to determine whether this membrane adds clinical advantages to the existing membranes for guided tissue regeneration. In vitro studies demonstrated that surface chemistry (hydrophilicity, surfaces with carboxyl groups) and nanotopographies play an important role on cell behavior and tissue integration based on macrophage polarization [6,29,30]. Ongoing experiments using large animal models are needed to check immunomodulation capacity and osteogenic differentiation.

## 5. Conclusions 

Zinc phosphate mineralized membranes, compared with non-mineralized membranes, have a significantly greater antibacterial effect, inhibiting bacterial colonization in vivo and so avoiding inflammation process and bone resorption. The Zn-Li-Ag alloy had a great biocompatibility, along with a resistance to corrosion and adequate mechanical properties. Zinc carbonate and zinc oxide were the main corrosion products. ZnBG-PLDLA membranes have shown appropriate mechanical properties such as strength and flexibility. The presence of ZnBG enhanced the osteogenic differentiation of the stem cells as confirmed by alkaline phosphatase, osteocalcin and sialoprotein, as well as the cellular mineralization. Precipitation of calcium and phosphate on the matrix surfaces was observed in zinc-loaded polymeric membranes, and these matrices were found to be non-toxic to cells. Even when the incorporation of metallic nanoparticles to the membranes did not exert cytotoxicity and seem to avoid adverse effects on the organisms, it has to be acknowledged that long-term human assays are scarce. Low amount of zinc oxide nanoparticles improves the bioactivity of the membranes. 

Experimental carboxylate membranes adsorbed higher concentration of tested proteins leading to major cells adhesion, proliferation and membrane integration into the surrounding tissue. The precipitation of calcium phosphate on the membrane surface was facilitated by membranes functionalized with carboxyl groups, which may lead to a rapid mineralization of the membrane. Incorporation of Zn in the membranes and their use for bone healing at rabbits calvarial provided the highest regenerative efficiency. Zn-loaded membranes promoted osteogenesis and enhanced biological activity, as mineralized and osteoid new bone with multiple interconnected ossified trabeculae, but without soft tissue invasion, appeared in close contact with the membrane. Zn-loaded Si-modified membranes showed the lowest ratio M1/M2, modulating the polarization of macrophages toward pro-healing phenotypes. Zn-HOOC-Si-Membranes obtained a balanced remodeling, enhanced biological activity and attained the best regenerative efficiency after angiogenesis and osteogenesis assessments. The bone-integrated Zn-HOOC-Si-Membranes may provide a novel therapeutic approach and can be considered as bioactive modulators, which increase M2 macrophages and so promoting bone repair. Quercetin-zinc complex (Zn + Q(PHt)) incorporated into polycaprolactone/gelatin nanofiber acted as a pharmacological agent for treating bone associated defects and guided bone regeneration.

## Data Availability

The data presented in this study are available on request from the corresponding author.

## Abbreviations

AFM	Atomic Force Microscopy
BMP-2	Bone morphogenetic protein-2
BPm	Bone perimeter
BS	Bone surface
BTh	Bone thickness
Ca/P	Calcium phosphate
Cq	Quantification cycle
ECM	Extracellular matrix
EDX	Energy-dispersive X-ray spectroscopy
FESEM	Field emission scanning electron microscopy
FGF	Fibroblast growth factor
Fn	Fibronectin
Fg	Fibrinogen
FTIR	Fourier transform infrared spectroscopy
GBR	Guided bone regeneration
HEA	Hydroxyethyl acrylate
HEMA	Hydroxyethyl methacrylate
LDH	Lactate dehydrogenase
MA	Methyl acrylate
MMA	Methyl methacrylate
Micro-CT	Microcomputed tomography
NM	Nano-structured membrane
OPG	Osteoprotegerin
OS	Osteoid surface
OS/TS	Percentage of osteoid surface
PLDLA	Poly-L-D, L-lactic acid
PLA	Polylactic acid
PMMA	Polymethylmethacrylates
PGA/TMC	Polyglycolic acid/trimethylene carbonate
PTFE	Polytetrafluoroethylene
PP	Plasma Proteins
SBFS	Simulated body fluid solution
SEM	Scanning electron microscopy
SiO_2_-NP	SiO_2_ nanoparticle
SRa	Surface nanoroughness
RANKL	Receptor activators of nuclear factor kappa-β ligand
rBMSCs	Rat bone marrow mesenchymal stem cells
TB	Toluidine blue
TEM	Transmission electron microscopy
TGF-β	Transforming growth factor-beta
THF	Tetrahydrofuran
TS	Total surface
XRD	X-ray diffraction
ZnBG	Zinc-containing bioactive glass
Zn-HAp	Zinc hydroxyapatite
Zn-NM	Zinc doped nano-structured membranes
